# Impact of Adhesive Layer Thickness on the Behavior of Reinforcing Thin-Walled Sigma-Type Steel Beams with CFRP Tapes

**DOI:** 10.3390/ma15031250

**Published:** 2022-02-08

**Authors:** Ilona Szewczak, Patryk Rozylo, Malgorzata Snela, Katarzyna Rzeszut

**Affiliations:** 1 Faculty of Civil Engineering, Lublin University of Technology, 36 Nadbystrzycka Str., 20-618 Lublin, Poland; m.snela@pollub.pl; 2 Department of Machine Design and Mechatronics, Faculty of Mechanical Engineering, Lublin University of Technology, 36 Nadbystrzycka Str., 20-618 Lublin, Poland; p.rozylo@pollub.pl; 3 Faculty of Civil and Transport Engineering, Institute of Building Engineering, Poznan University of Technology, 5 Marii Skłodowskiej-Curie Str., 60-965 Poznań, Poland; katarzyna.rzeszut@put.poznan.pl

**Keywords:** adhesive connection, cold-formed steel beam, reinforcement method, composite tapes

## Abstract

This paper presents selected issues related to the reinforcement of steel element cold-formed with CFRP tapes. The first section of the paper is a review of source literature and a presentation of the basic information on cold-formed thin-walled steel elements and CFRP composite materials, stressing the advantages and disadvantages of using them to reinforce steel structures. Next, the authors present original research on reinforcing bent thin-walled sigma-type steel beams using adhesive CFRP tapes. Reference beams with a cross-section of Σ200 × 70 × 2 and a length of 3 m, reinforced with CFRP tape, were tested in the four-point bending scheme. Then, the paper discusses a developed numerical model that is consistent with the subject matter of the laboratory tests. The developed numerical model was prepared to represent the failure of the connection between the beam and the composite tape. This was followed by a number of numerical analyses in order to determine the optimum adhesive layer that would allow us to achieve the maximum reduction of the displacements and strains in bent thin-walled sigma-type beams. Three thicknesses of the SikaDur adhesive layer were analyzed in the study. Based on the analyzes, it was found that the increase in the thickness of the adhesive layer slightly reduced the strain and displacement in the beams, but caused a significant decrease in the load value, at which damage appeared in the glued joint.

## 1. Introduction

Forming main load-bearing elements of building structures from cold-formed sections enables us to not only satisfy requirements in terms of load-bearing capacity, durability, reliability, aesthetics and functionality, but also satisfy requirements associated with the concept of sustainable construction, under the strategy of sustainable economic growth, owing to responsible consumption and production that takes into account the impact of construction on climate change. Compared to traditional structural solutions, cold-formed elements have one of the highest ratios between the strength and the weight of the material used for manufacturing. 

However, it should be noted that their wide structural application range may also generate a potential need for reinforcement, arising from, among others, design mistakes or increased external loads relative to the design. Unfortunately, structures made of thin-walled steel elements are characterized by limited reinforcement opportunities. Due to the very thin walls of their cross-section, the thickness of which amounts from 1 mm to 3 mm, it is virtually impossible to weld them, and the introduction of connections using mechanical fasteners is also significantly hindered. As a result, it seems necessary to search for an effective and easy method of reinforcing these beams. One of such methods may be the application of composite materials (FRP—fibre-reinforced polymers/plastics), which are based on high-strength non-metallic fibres embedded in an epoxy matrix, e.g., CFRP, GFRP and AFRP tapes, made of carbon, glass and aramid fibres, respectively. These tapes are glued to the structure, ensuring rapid and effective strengthening, virtually without limiting operational continuity.

When conducting literature studies on reinforcing thin-walled steel structures using CFRP materials, one can find a very limited number of publications, most of which focus on studying compressed elements. For example, Bambach, M.R. [1] described a study of thin-walled steel elements of a square section reinforced with CFRP on the outside. The experiment was conducted at quasi-static axial compression, in order to determine the impact of the applied reinforcement on the increase in compressive strength. It was also conducted for an axial impact. The work effort focused on testing the impact of reinforcement relative to dynamic axial compression and associated impact resistance indicators (against axial compression). Park, J.W. et al. [2] studied the behaviour of axially compressed thin-walled square steel columns that were reinforced with CFRP. In total, their research involved eight samples. Based on the obtained results, the researchers concluded that reinforcing the entire cross-section with three CFRP layers with fibers, arranged transversely to the force direction, enabled the increase of the maximum load by 33%. They also developed FEM models that confirmed the increased rigidity of reinforced columns. Imran, M. et al. [3] also studied axially compressed thin-walled columns with a square cross-section. They used a reinforcement in the form of CFRP mats glued around the entire section, and demonstrated that strengthened samples were able to transfer a load that was 2.6 times higher, relative to non-reinforced samples, than the compressive force, until first local buckling cases appeared. In order to conduct more comprehensive analyses, they also developed a numerical model in Abaqus software. The behaviour of axially compressed cold-bent square sections reinforced with CFRP is described in [4]. Within the study, composite material was glued in layers around the cross-section, so that the fibres in the first layer were perpendicular to the load direction, and parallel in the second layer. The obtained results indicated that the application of CFRP significantly delayed local buckling, and elastic buckling strength was increased even four-fold. The information contained in the paper is an extension of similar studies described by Bambach, M.R. et al. [5]. The authors described a design method for the determination of the axial compressive strength of elements circumferentially reinforced with CFRP, the results of which indicated compliance with 45 samples subject to laboratory testing. As emphasized by Bambach, M.R. [1], the motivation behind reinforcing thin-walled steel structures using CFRP in the case of axial compression [6] is for enabling the control of buckling strains and ensuring increased compressive strength. Silvestre, N. et al. [7], presented a study involving nineteen samples taken from a thin-walled C-section subjected to compression. Seven various CFRP material locations and two fiber arrangements were analyzed. The objective was to study the impact of CFRP on the operation of the element and to determine limit loads that were identified by the appearance of various failure mechanisms. Based on the obtained results, it was concluded that reinforcing the entire C-section enabled them to improve the maximum load by 20% under the test conditions. Tests were used to develop a detailed model in the Abaqus software. A year later, in paper [8], the same authors suggested expanding the design guidelines in Eurocode 3 and AISI-DSM with recommendations regarding the design of compressed thin-walled C-section elements reinforced with CFRP. As it has already been stated, a majority of the cited papers focused on studying elements subjected to compression, whereas in the case of bent thin-walled beams, this number is limited [9,10].

## 2. Thin-Walled Structural Elements

Thin-walled bars are characterized by a specific ratio of geometric dimensions. Section wall thickness is much smaller than its transverse dimensions, and its length is much higher than the cross-section dimensions. In engineering practice, a thin-walled bar is an element, the geometrical dimensions of which, such as thickness, width and length, differ by an order of magnitude. According to this definition, thin-walled bars include almost all sections used in steel structures, both hot-rolled, welded, riveted and cold-formed. Vlasov, the creator of the theory on thin-walled open-section bars, is more precise in describing a thin-walled bar. He states that a bar may be considered thin-walled if the wall thickness is at least eight times lower than the longest distance measured along the center line between two extreme points located on the bar cross-section contour. This distance should be at least eight times lower than the bar length. Detailed guidelines regarding the limits of transverse dimension values for thin-walled elements are indicated in [11,12,13].

The abundance of available thin-walled section shapes provides steel structure designers not only with wide possibilities of element selection but, as noted by Oswald, M. et al. [14], at the same time contributes to the creation of new engineering issues associated with the interaction of global and local forms of stability loss. Paradoxically, the more time and attention are devoted to laboratory and numerical analysis of thin-walled steel structures, the more interesting research problems arise. This is why numerous research centers are still intensively working on the research issues concerning thin-walled steel structures.

## 3. CFRP Tapes

Composite materials (FRP—fibre-reinforced polymers/plastics) are materials that are based on high-strength non-metallic fibres, embedded in an epoxy matrix [15]. There are currently three types of composite materials commonly used in the construction industry. They differ in terms of the fibre material. These are: CFRP (carbon fibre-reinforced polymer), GFRP (glass fibre-reinforced polymer) and AFRP (aramid fibre-reinforced polymer). CFRP tapes and mats with matrices based on carbon fibres are the composite materials most commonly used to reinforce building structures, which results from very good strength parameters exhibited by carbon fibres relative to other materials. Composite material manufacturers declare a very wide scope of potential applications for CFRP tapes [16]. They can be used wherever reinforcing an existing structure is required due to increased operating loads (e.g., changed facility purpose), or the appearance of new loads have arisen, e.g., installation of heavy devices. They are also applied in the case of a required reinforcement of load-bearing elements in structures damaged due to corrosion, fire, or mechanical action, as well as in the case of a need to improve their operating conditions, i.e., limit strains, increase fatigue capacity, reduce stress, or reduce crack opening. The application of CFRP tapes also enables adapting a structure to a potential change of the static schema, e.g., due to removed walls or columns. CFRP tapes can be very useful in the case of a need to improve structural load-bearing capacity in the event of special loads, such as explosions or impacts caused by vehicles, and also in the case of design or execution errors. The main properties of CFRP composites depend on the type and orientation of carbon fibres, the parameters of the used epoxy resin, and its percentage share in the finished material, as well as drying conditions [17]. The advantages and disadvantages of CFRP tapes are described in detail in [10].

## 4. Reinforcement Technology for Thin-Walled Steel Beams

Three very important issues should be considered when commencing the reinforcement of steel structures using CFRP tapes. These are the location of the CFRP tape, correct selection of tape length, and the thickness of the adhesive layer.

Available research reports include studies on the correct selection of the CFRP tape bond length (Lz) in the case of reinforcing hot-rolled steel elements [18] or bridge girders [19]. However, the CFRP tape bond lengths they recommended were of different values. Furthermore, it is hard to find an unambiguous definition of an effective length in the source literature, since it is interpreted in many ways. For example, based on the conducted studies involving a joint between high-strength steel pipes, the authors of [20] determined an effective bond length for CFRP tapes (Lz) equal to 75 mm. The authors of [19] published test results involving steel girders damaged by bending. For the purposes of their research, they defined the bond length as the shortest length that enabled transferring the largest load onto a CFRP tape. Based on studying five adhesive types, they determined this value at approximately 203 mm. The authors of [21] concluded that 98% of the forces are transferred at a distance of 100 mm from the end of the CFRP tape. The author of [22] analyzed three CFRP tape bond distances: 65 mm, 165 mm and 265 mm. He confirmed that almost all forces transferred from a steel beam onto a CFRP tape occur at a distance of approximately 70 mm from its end, and only in the case of a bond length equal to 265 mm did he observe yielding of the steel prior to detachment of the CFRP tape. Such a bond length provides the best reinforcement system strength. In the case of determining the bond length for a CFRP tape used to reinforce thin-walled elements, Bastani, A. et al. [23] used a bond length equal to 31 cm when reinforcing T-section beams; however, it was not supported by any analyzes. Rzeszut, K. et al. [24], based on numerical analyses conducted in Abaqus software, concluded that an effective bond length of 70 mm was sufficient; however, they only analyzed strains in bent thin-walled beams. 

**Figure 1:**
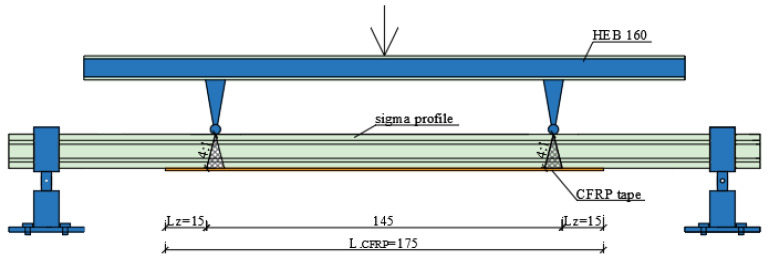
Scheme defining the effective bond length of CFRP tapes. (Unit: cm).

It should be stressed that there is a need to expand this analysis with the impact of the bond length on beam displacement in order to formulate clear conclusions. Based on current recommendations in terms of CFRP tape bonding, this paper adopted an effective length in accordance with the diagram [22] shown in Figure 1, where Lp is prop spacing, LCFRP is the CFRP tape length, and Lz is the CFRP tape effective bond length.

Another very important issue is the selection of a proper location for CFRP tapes. While in the case of concrete or reinforced concrete structures it is obvious that tapes are placed in the tensile zone, when it comes to cold-formed thin-walled steel structures, this is not so straightforward. For example, sigma-type thin-walled beams studied under a four-point bending system undergo bending or twisting due to a shifted center of gravity. This requires making a choice as to whether we primarily want to limit strains or displacements in the bottom and upper sections. Appropriate tape location should be adopted based on this decision.

The next important issue concerns the selection of the optimum adhesive layer thickness. One of the few studies concentrating on the selection of the optimum adhesive layer thickness is described in [22]. Its author analyzed the effectiveness of reinforcing hot-rolled I-section steel beams subject to bending. The beams were reinforced with a CFRP tape using three adhesive layer thickness (0.65 mm, 1.3 mm and 1.75 mm). The highest destructive force value was achieved by beams where the adhesive thickness was 1.3 mm. On the other hand, the lowest destructive force value was achieved by samples with an adhesive thickness of 1.75 mm. The authors of this study did not encounter such research concerning the reinforcement of cold-formed thin-walled steel beams, which was the impetus behind addressing this issue. Therefore, laboratory tests were carried out, in which steel sigma cross-section beams, reinforced with CFRP tape glued to the beams with a 1.3 mm thick adhesive layer, were tested in the four-point bending scheme. In order to verify whether the thickness of the adhesive bond layer was optimal, it was decided to develop a numerical model that was subjected to validation based on conducted laboratory testing, followed by conducting a series of numerical analyses for other adhesive layer thicknesses (0.65 mm and 1.75 mm). Based on the analyses, it was found that the increase in the thickness of the adhesive layer slightly reduces the strain and displacement in the beams, but causes a significant decrease in the load value, at which damage appears in the glued joint.

## 5. Materials and Methods

For the purposes of this article, the laboratory testing involved five sigma-type thin-walled 200 mm high beams. Their flange width was 70 mm, wall thickness 2 mm, and span 280 cm. Beams were made of S350GD steel grade with strength properties as follows: Young’s modulus E = 201.8 GPa; Poisson’s ratio ν = 0.28; and the yield point of steel fy = 418.5 MPa. Material characteristics were determined on the basis of laboratory coupon tests. The tests were carried out on five samples, cut out from sigma profiles of the size and shape compliant with the requirements of PN-EN ISO 6892-1: 2009. A biaxial extensometer was used to measure the longitudinal and transverse deformation of the samples.

Two beams were tested without reinforcement (B1R, B2R), and three beams were reinforced with a CFRP tape placed on the upper flange (B1G, B2G, B3G). Sika CarboDur S carbon fibre, 1.2 mm thick and 50 mm thick tapes were used as reinforcement. Material tests enabled determining Poisson’s ratio ν = 0.308 and Young’s modulus E = 165 GPa, which was mentioned in [9,10].

CFRP tapes were glued to the beams using a SikaDur^®^-30 glue (Warsaw, Poland). Basic strength characteristics of the adhesive, specified in the manufacturer’s material sheet, included minimum compressive strength after 7 days equal to 75 MPa, compressive elasticity modulus of 9600 MPa, minimum tensile strength after 7 days of 26 MPa, minimum steel peel strength after 7 days of 21 MPa, minimum shear strength of 16 MPa, and shrinkage of 0.04%. The reinforced beams were subjected to loading after a minimum of 7 days from gluing the CFRP tape in order to obtain the full strength of the glued joint, in accordance with the manufacturer’s recommendation.

**Figure 2:**
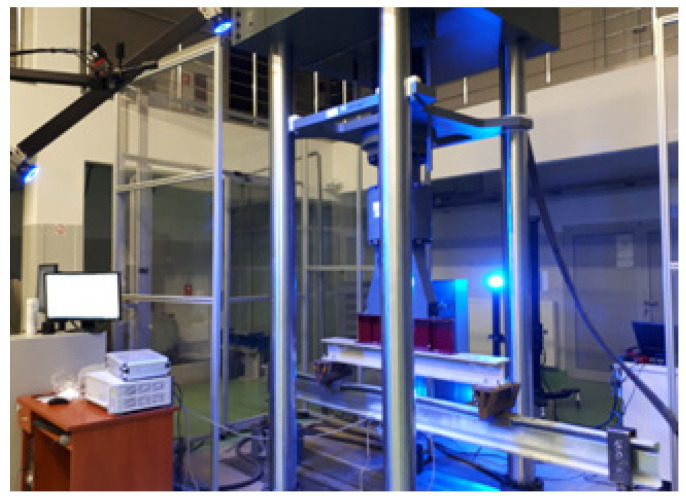
Laboratory stand.

**Figure 3:**
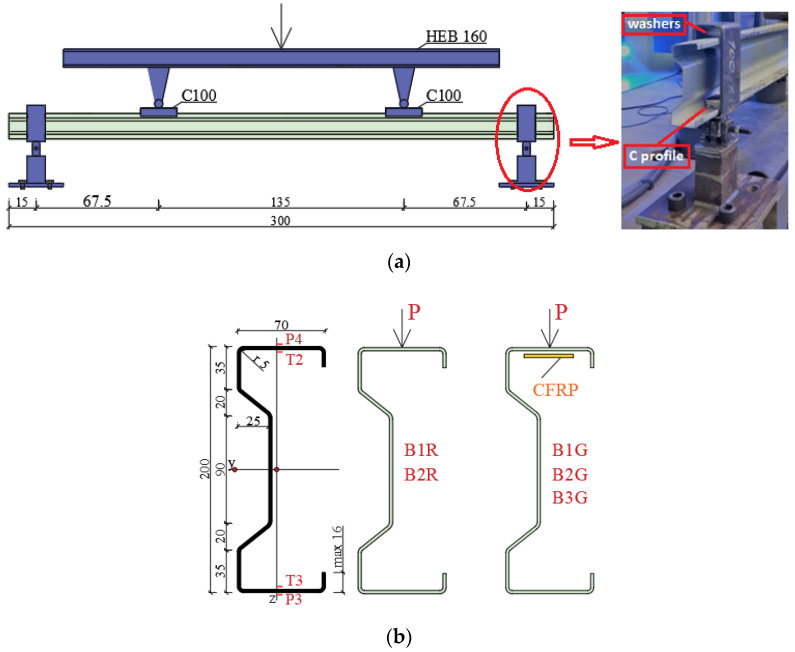
(**a**) The scheme of laboratory stand; (**b**) cross-section of the tested beam, the location of the CFRP tape and layout of displacement measurement points (P) and electrofusion strain gauges (T).

The adhesive was prepared in accordance with the manufacturer’s instructions, and reinforced beams were subjected to load after a minimum of 7 days from gluing the CFRP tape, in order to obtain full adhesive bond strength. Adhesive thickness was 1.3 mm. The CFRP tape length was 175 cm. All beams were subjected to testing under a four-point bending system. The laboratory stand photo is shown in Figure 2. The Zwick Roel (Zwick Roell GmbH & Co., KG, Ulm, Germany) testing machine at the Construction Laboratory of the Lublin University of Technology was used in order to perform experimental tests. Figure 3 shows a laboratory stand diagram, studied beam cross-section, CFRP tape location, the distribution of the analyzed measuring points for displacement (P), and electrofusion strain gauges (T).

In the course of the test, the beams were bent and twisted. As a result, the Tritop and Aramis systems were used to correctly measure the displacements. Vertical displacements were measured at points P3 and P4 located in the middle of the beam span. Strains were measured using electrofusion strain gauges (type TENMEX TFs-10 with 120 Ω ± 0.2% resistance) marked T2 and T3, in the middle of the beam span. 

Laboratory tests were conducted until the beams were completely destroyed (in the case of non-reinforced beams) or until the CFRP tape was detached. Therefore, the criterion of failure was the load, exceeding which the CFRP tapes detached, amounting to 25 kN.

## 6. Numerical Analyses

**Figure 4:**
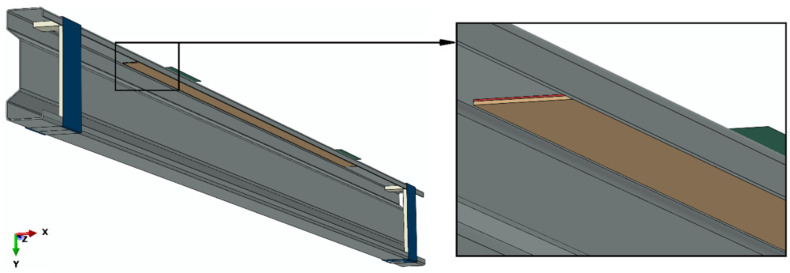
Numerical model.

Numerical simulations were carried out on the basis of static calculations. As part of the numerical simulations conducted using the finite element method, a comprehensive discrete model of the structure was prepared, containing the main element of the system, i.e., a sigma steel profile with the dimensions of 200 × 70 × 2 mm and a length of 3000 mm, as well as all components such as supports, stiffening elements in the area of U-shaped supports, composite tapes made of CFRP material, adhesive bonds between the beam and the composite tape, and non-deformable plates transferring loads to the beam. Between the beam and the composite strip where the adhesive layer was located were Tie-type permanent fastener interactions. All elements of the tested system were mapped with due care, so that the numerical simulations were a faithful reflection of the experimental tests. During the numerical simulations conducted with the use of Abaqus^®^ (2021) (Dassault Systemes Simulia Corporation, Velizy Villacoublay, France), discrete models were prepared, which allowed for further comparison of the numerical simulations results with the results of experimental tests. Details of mechanical properties used in the numerical simulation are presented in [9,10]. For the discrete model of the main structure of the sigma steel beam, solid finite elements of the C3D20R type were used, as characterized by the second-order shape function, and the twenty-node-type with reduced integration. The composite tape and U-shaped stiffening elements were modelled using S4R type shell finite elements, as characterized by the first-order shape function, and the four-node-type with reduced integration. The supports and plates transferring loads to the beam were prepared with the use of non-deformable elements of the R3D4 type. The adhesive layer between the composite tape and the corresponding surface of the sigma beam was modelled by COH3D8 cohesive finite elements. The numerical model (Figure 4) was prepared so that the sigma beam (gray) was placed between the non-deformable supports (blue), where channel-shaped stiffening elements (light gray) were inserted in the beam profile adjacent to the supports. Adjacent to the upper flange of the beam were non-deformable plates (green), constituting the loading elements, while both the aforementioned supports and stiffening elements, as well as the non-deformable plates, remained in contact with the sigma beam. Moreover, a composite tape (beige) was attached to the beam through an adhesive bond (red) between the tape and the beam. The system prepared as described above was subjected to numerical analysis (Figure 4).

**Figure 5:**
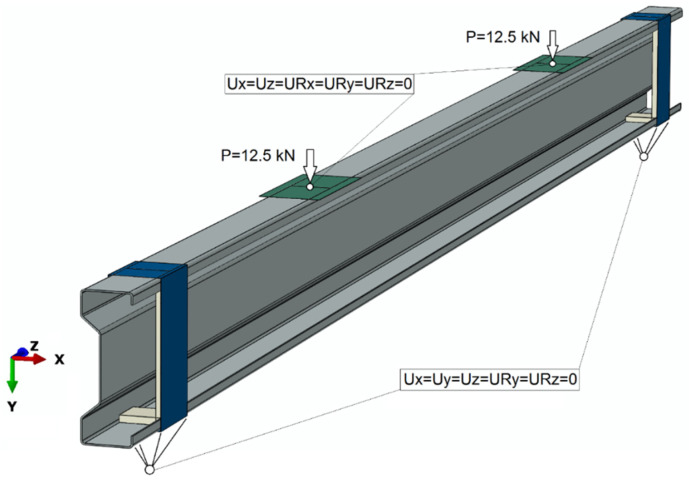
Boundary conditions.

As part of the numerical simulations, three different analysis cases were considered, where the entire system remained unchanged, and only the thickness of the adhesive layer was changed. Initially, the thickness of the adhesive layer was taken as equal to 0.65 mm, and then the results for the thickness of the adhesive layer of 1.3 mm and 1.75 mm were also analyzed. Thus, the impact of the adhesive layer thickness on the stability of the structure and strength parameters was analyzed. Numerical tests were carried out maintaining contact in the tangential and normal directions, with the coefficient of friction between the contacting elements at the level of 0.2. For the numerical model, the MAXS maximum nominal stress criterion was used to simulate the damage initiation of the cohesive bond while the damage evolution was based on the energy criterion. The use of failure criteria allowed further simulation of the adhesive joint failure (in the case of the Abaqus program, the SDEG parameter was responsible for this). In the numerical tests, the necessary boundary conditions were provided, which were defined as shown in Figure 5.

Boundary conditions were defined at reference points that were coupled with selected elements of the tested system (Figure 5). The boundary conditions were defined on the basis of receiving certain degrees of freedom of selected system elements, which enabled the mapping of the actual behavior of the sigma beam structure. The loads of 12.5 kN were assigned to both non-deformable plates transferring loads to the main beam structure, which corresponded to the actual load carried out through experimental tests.

**Figure 6:**
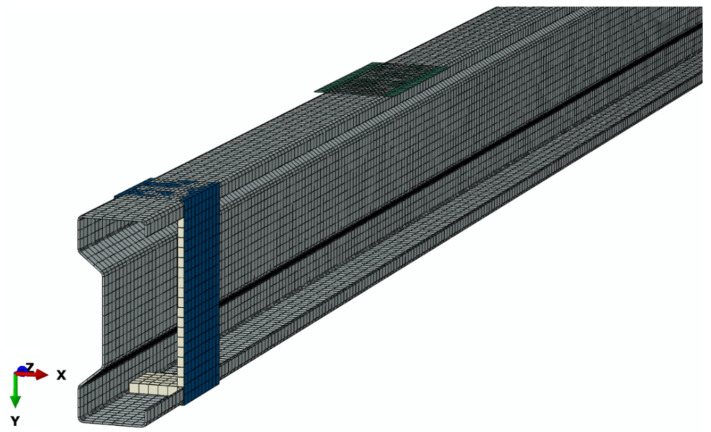
Discrete model—part of the analyzed structure.

Within the framework of the developed numerical model, discrete models of individual components of the analyzed research system were prepared. The discrete model of the tested system was characterized by the presence of 37,788 computational nodes for 19,463 finite elements (Figure 6).

In the case of the applied components of the numerical model, material models adequate to the material parameters obtained in the experimental tests were used. For the composite tape, a special material model with orthotropic characteristics was prepared, where the tape was made of one layer of composite material. The adhesive layer was based on a material model using cohesive elements in order to simulate the failure of the adhesive joint. In the case of the main beam structure, as well as U-shaped stiffening elements, an isotropic material model based on steel properties (presented in previous chapters) was used, while the objects constituting the supports and elements transferring loads to the structure were non-deformable elements, for which no material model was defined within the framework of the numerical simulations. 

## 7. Results of Laboratory Tests and Numerical Analyses

**Figure 7:**
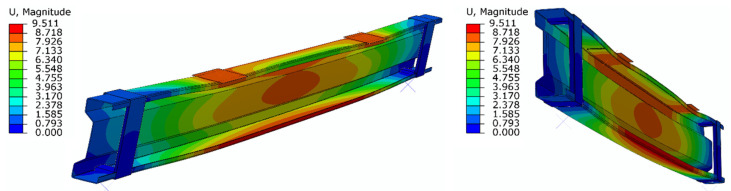
Displacement distribution—beam with 0.65 mm adhesive layer.

During laboratory tests, in the majority of the beams, the CFRP tapes detached from the beams after exceeding the load of 25 kN. Therefore, the load of 25 kN was considered as a destructive force, and at this load level, the results of displacements and strain obtained in individual samples during laboratory tests and in subsequent numerical models were analyzed. Table 1 shows the values of displacements and strain of individual models and laboratory samples with a load value of 25 kN. The table also includes the results obtained by reference beams in order to present the benefits of using CFRP tape reinforcement. Figure 7 presents an example of the displacement distribution obtained by means of the numerical model, in which the thickness of the adhesive layer was equal to 0.65 mm.

**Figure 8:**
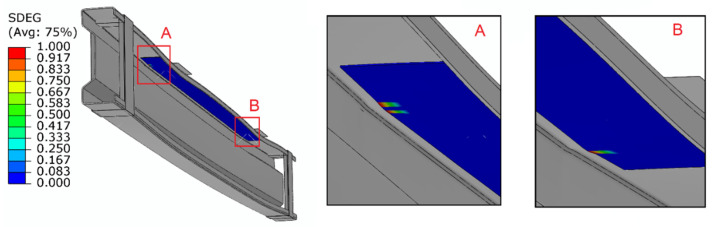
Result of damage to the adhesive bonding: (**a**) front side of adhesive; (**b**) back side of adhesive.

Furthermore, as part of the numerical tests, the scope of damage within the adhesive layer was also analyzed. For numerical simulations, the SDEG (stiffness degradation) parameter was used in order to assess the adhesive stress distribution, which allowed for the description of the adhesive layer damage. When this parameter was set to 1, it meant that this connection in a given area was damaged. In the case of the conducted research, it was observed that the adhesive layer was initially damaged adjacent to the areas to which the load was applied. It was estimated that the adhesive layer began to fail at a load of 26.94 kN for a 0.65 mm adhesive layer, 24.4 kN for a 1.3 mm adhesive layer and 23.7 kN for a 1.75 mm adhesive layer. For the purposes of better visualization of the damage to the adhesive layer, the composite tape that lied directly on the adhesive layer was not involved in the visualization (see Figure 8).

## 8. Analysis of the Results

The developed numerical model showed a high degree of convergence with the results of laboratory tests carried out for beams reinforced with CFRP tape glued with a 1.3 mm thick adhesive. The satisfactory convergence concerns both the values of strain and the displacements observed for the load value of 25 kN, as well as for the moment of separating the tape from the beam at the glue–steel bonding surface. In laboratory tests, for a beam reinforced with CFRP tape in the upper flange, tape detachment was observed at a load value of 25.4 kN, but it is apparent that the first damage in this connection appeared much earlier.

Based on laboratory tests, it was found that use of CFRP tapes placed on the upper flange allowed a reduction in the strain in the top and bottom flange by 14.3% and 3.6 %, respectively, and a reduction in the vertical displacements of the top flange by 11.8% and the bottom flange by 11.2%, with respect to the reference beams, at the load level of 25 kN. The obtained results refer to the 1.3 mm adhesive layer thickness.

In the case of numerical analyses, the increase in the thickness of the adhesive layer caused a decrease in the strain in both flanges of the beam and in the vertical displacements in the top flange. As for the vertical displacement in the bottom flange, for the thickness of the adhesive layer of 1.3 mm, an increase in the vertical displacement in relation to the numerical model, where the joint thickness was 0.65 mm, was noted. It should be emphasized, however, that in the case of strain, its reduction was insignificant, and in the case of displacements, it did not exceed 0.3 mm. Therefore, much more attention should be paid to the load values at which the first damage in the individual models appeared in the adhesive connection. As the thickness of the adhesive layer increased, the value of the load at which the first defects appeared in the adhesive layer decreased.

## 9. Conclusions

The numerical model developed for the purposes of this study indicated high compliance with the laboratory tests carried out.

Based on the conducted numerical analyses, it was found that:

- With the increase in the thickness of the adhesive layer connecting the thin-walled steel sigma beam with the CFRP tape, the strain in the upper and lower beam flanges decreased slightly, as well as the vertical displacements in the upper compartment (not more than 0.3 mm);
- With the increase in the thickness of the adhesive layer, the value of the load at which the first defects appeared in the adhesive layer decreased (the difference in the obtained load values between the smallest and the largest thickness of the adhesive layer was 3.24 kN—it is a significant difference at the load value of 25 kN);
- It can be assumed that the value of the load at which the first failure occurs in the adhesive layer will be directly related to the force at which the failure of the beams will occur.

Based on the above considerations, it can be concluded that in the case of reinforcing thin-walled steel sigma beams with CFRP tapes, the most advantageous method is to use an adhesive layer with a thickness of 0.65 mm, which the authors of the study intend to check in subsequent laboratory tests. It was proved that the results of numerical simulations, were positively verified by experimental studies [25,26,27,28].

## Figures and Tables

**Table 1:** Strain and displacements of individual models and laboratory samples with a load value of 25 kN.

Sample	Strain [×10^−6^]				Vertical Displacements (mm)			
	T2		T3		P3		P4	
Laboratory tests								
B1R	741	756.5	968	956	11.6	11.6	10.26	10.26
B2R	773		944		-		-	
B1G	618	648	908	921.7	10.15	10.3	9.1	9.05
B2G	677		933		10.7		8.55	
B3G	649		924		10.1		9.5	
Numerical anlayses								
Model with adhesive layer thickness equal to								
0.65 mm	657		892		9		8.33	
1.3 mm	654		891		9.04		8.13	
1.75 mm	652		887		8.96		8.05	

## Data Availability

Data is contained within the article.
